# Impact of Gastrointestinal Digestion In Vitro Procedure on the Characterization and Cytotoxicity of Reduced Graphene Oxide

**DOI:** 10.3390/nano13162285

**Published:** 2023-08-09

**Authors:** Óscar Cebadero-Domínguez, Leticia Diez-Quijada, Sergio López, Soraya Sánchez-Ballester, María Puerto, Ana M. Cameán, Angeles Jos

**Affiliations:** 1 Area of Toxicology, Faculty of Pharmacy, Universidad de Sevilla, 41012 Seville, Spain; ocebadero@us.es (Ó.C.-D.); ldiezquijada@us.es (L.D.-Q.); camean@us.es (A.M.C.); 2 Department of Cell Biology, Faculty of Biology, Universidad de Sevilla, 41012 Seville, Spain; serglom@us.es; 3 Packaging, Transport and Logistic Research Institute, Albert Einstein, 1, Paterna, 46980 Valencia, Spain; soraya.sanchez@itene.com

**Keywords:** in vitro digestion, reduced graphene oxide, 3D model, cytotoxicity, internalization

## Abstract

The growing interest in graphene derivatives is a result of their variety of applications in many fields. Due to their use, the oral route could be a potential way of entrance for the general population. This work assesses the biotransformation of reduced graphene oxide (rGO) after an in vitro digestion procedure (mouth, gastric, intestinal, and colon digestion), and its toxic effects in different cell models (HepG2, Caco-2, and 3D intestinal model). The characterization of rGO digestas evidenced the agglomeration of samples during the in vitro gastrointestinal (g.i.) digestion. Internalization of rGO was only evident in Caco-2 cells exposed to the colonic phase and no cellular defects were observed. Digestas of rGO did not produce remarkable cytotoxicity in any of the experimental models employed at the tested concentrations (up to 200 µg/mL), neither an inflammatory response. Undigested rGO has shown cytotoxic effects in Caco-2 cells, therefore these results suggest that the digestion process could prevent the systemic toxic effects of rGO. However, additional studies are necessary to clarify the interaction of rGO with the g.i. tract and its biocompatibility profile.

## 1. Introduction

Graphene nanomaterials are increasingly being used in the industry due to their distinctive characteristics that make them promising nanomaterials for different applications such as electronics [1], optics [2], material sciences [3], physics [4], gene therapy, biosensors, phototherapy, drug delivery, or tissue engineering [5,6]. Graphene is an allotrope of carbon with its atoms arranged in a benzene-ring structure in a two-dimensional hexagonal lattice with unique properties that make it different from other allotropes of carbon [7]. The group of graphene-related materials (GRM) includes few-layer graphene (FLG), ultrathin graphite, graphene oxide (GO), reduced graphene oxide (rGO), graphene nanosheets, graphene nanoribbons, and graphene quantum dots (GQD) [8].

Human exposure to graphene is likely due to the increased use of GRM in recent years. Inhalation is considered the main route of exposure to GRM in humans, and therefore most published studies, both in vitro and in vivo, are focused on the respiratory tract (lungs) [9]. Moreover, the oral route could be a potential way of entrance, because of the use of GRM in the food industry, such as food packaging, medicine, agriculture, and contamination of water and food, and also indirectly due to the ingestion of inhaled material [9]. In any case, data regarding human exposure assessments are scarce and they refer mainly to occupational exposures [9,10].

rGO is a graphene derivative that is obtained by the reduction of GO by thermal or chemical methods. The aim of reducing oxygen functional groups of GO is to produce materials with properties close to pristine graphene [11]. This procedure increases rGO hydrophobicity in comparison to GO and reduces surface charge and water dispersibility [12], which improves its ability to be employed into many applications [13]. Moreover, rGO can be more suitable than other graphene derivatives for the food packaging industry, due to its exceptional ability to limit oxygen permeation and improve shelf life of foods, increasing the antioxidant activity of packaging materials [14,15].

Despite the interest of rGO food-related applications, the number of studies that have assessed the effect that the human digestion process has on GRM is scarce [16,17,18,19], and only one has evaluated this process on rGO [19]. Main findings of these reports are provided in Table S1 (Supplementary Material). Nevertheless, none of these studies includes the impact of the colonic phase, which is necessary to mimic the passage through the entire human digestive tract and to simulate a more accurate human exposure scenario [20]. The use of simulated gastrointestinal (g.i.) digestion is extensively used in different fields of food and nutritional science because conducting human assays is usually costly, resource intensive, and ethically disputable [21]. Furthermore, in vitro models of oral digestion mimic the conditions of the g.i. tract (including mouth, stomach, and gut) [22] and it is representative of the real in vivo environment. An important factor to consider is that the amount ingested does not always coincide with the amount available to organisms, as some metabolic processes can alter the fraction of the available contaminant [20]. In addition, some GRM can be degraded in the stomach and then precipitate under intestinal conditions [23]. As ingested GRM go through the g.i. tract, they are subjected to several conditions (temperature, pH, ionic strength, salts, digestion time), digestive enzymes (amylase, mucin, pancreatin, pepsin), and colonic bacteria that could change the physicochemical properties and the toxicological profile of these materials [21,24].

Therefore, the objective of this study was to investigate the effect of an in vitro digestion model including salivary, gastric, duodenal, and colonic phases on rGO and the potential impact on its physicochemical properties and cytotoxicity. For this purpose, rGO was characterized in all digestion phases. Digested rGO (duodenal phase) was used to assess cytotoxicity, cell uptake, and inflammatory response in a representative in vitro model of liver (HepG2 cell line), since it has been proven that GRM may be absorbed and distributed throughout the organism to the hepatic system [23], and in duodenal and colonic phases in Caco-2 cells as it is a well-characterized model of the intestinal epithelium [25], commonly used in toxicity studies. In addition, cytotoxicity of rGO (undigested and duodenal phase) was also assessed in a three-dimensional (3D) human intestinal in vitro model (EpiIntestinalTM).

## 2. Materials and Methods

### 2.1. Chemicals and Reagents

rGO was purchased from Sigma-Aldrich (Madrid, Spain). Chemicals for the in vitro digestion model were obtained from Sigma-Aldrich. Bacteria culture medium (de man, Rogosa, Sharpe) was supplied by Oxoid (Madrid, Spain), and Microbiology Anaerocult^®^ A, used to generate an oxygen-depleted and CO_2_-enriched atmosphere, by Merck (Darmstadt, Germany). Culture medium, cell culture reagents, and fetal bovine serum (FBS) were provided by Gibco (Biomol, Sevilla, Spain). Reagents for the Bio-Plex ProTM human cytokine assay kit were obtained from Bio-Rad Laboratories (Hercules, CA, USA).

### 2.2. In Vitro Digestion Model

The assay was carried out according to Diez-Quijada et al. [20] and EFSA recommendations [22]. Three samples of rGO (50, 100, and 200 µg/mL) were dispersed in 15 mL Milli-Q water and sonicated 1 h to reduce particle agglomeration. rGO samples were mixed with 14 mL of artificial saliva and 171 mL of water and shaken for 30 s to simulate the oral phase. Afterward, 0.25 g pepsin was added and the pH was corrected to a value of 2 with the aim of activating the enzyme. Later, the mixture was incubated for 2 h at 37 °C in an orbital shaker (250 rpm). In the duodenal phase, the pH was increased to 6.5 and 2.25 g of pancreatin and bile salts mix were incorporated into the mixtures. Samples were shaken again for 2 h at 37 °C (250 rpm). In the last phase, duodenal intestinal fluids were incubated with a mixture of lactic acid bacteria (LAB) at 1 × 10^8^ CFU/mL for 48 h at 37 °C under anaerobic conditions (5% CO_2_/95% air) to mimic the colonic environment. The selected bacterial strains used are a suitable representation of the real conditions in humans. LAB include many bacterial genera, such as *Lactobacillus (Lb.) casei* CECT 4180, *Lb. casei rhamnosus* CECT 278T, *Lb. plantarum* CECT 220, *Lb. delbur sub bulgaricus* CECT 4005, *Lb. salivarus* CECT 4305, *Lb. johnsoni* CECT 289, *Bifidobacterium breve* CECT 4839T, and *B. bifidum* CECT 870T. All of them were obtained from the Spanish Type Culture Collection (CECT, Valencia, Spain) in sterile 18% glycerol [20].

### 2.3. Characterization

At the end of each phase, an aliquot of each sample was taken to analyze and characterize the rGO samples by Fourier transform infrared spectroscopy (FTIR), Z potential, and scanning electron microscopy (SEM). In addition, in the duodenal phase, samples were collected at 5, 15, 30, and 60 min. Fourier transform infrared (FTIR) spectroscopy experiments were carried out in a Bruker Tensor 27 IR spectrometer (Bruker, Germany) in the range of 3800–600 cm^−1^ using the attenuated total reflectance mode at a resolution of 4 cm^−1^ and 64 scans. Background spectra were collected before each series of experiments to eliminate any interference from the environment. Z potential was measured by Malvern, Zetasizer Nano ZS available in the Functional Characterization Service (CITIUS). Samples for the SEM were deposited on pins with double-sided carbon adhesive tape. Images were obtained using the Zeiss EVO microscope at 10 KV available at the Microscopy Service (CITIUS).

### 2.4. In Vitro Cell Models

The human cell lines HepG2 and Caco-2 were maintained as described in Houtman et al. [26]. EpiIntestinal^TM^ (SMI-100) was obtained from MatTek Corp. (Ashaland, MA, USA). This is a 3D intestinal model used for toxicity testing as it shows more similarity to human small intestine tissue. It is formed of villi structures, brush borders, and columnar epithelium. The 24 EpiIntestinal tissues were transferred to two 12-well plates with SMI-100 medium. The plates containing the tissues were equilibrated into a humidified incubator overnight at 37 °C, 5% CO_2_ before the exposure.

### 2.5. Uptake and Cytotoxicity

To check the digested graphene uptake by HepG2 and Caco-2 cells, the cultures were exposed to 100 μg/mL rGO (undigested and duodenal phase in HepG2, and duodenal and colonic phases in Caco-2) for 24 and 48 h according to the procedure of Cebadero-Domínguez et al. [27].

For the cytotoxicity tests, HepG2 cells were exposed to different concentrations of undigested rGO (0–250 µg/mL) and digested rGO after the duodenal phase (50, 100, and 200 µg/mL). Caco-2 cells were exposed to undigested (200 µg/mL) and digested rGO after duodenal and colonic phases (50, 100, and 200 µg/mL) for 24 and 48 h. The digestas taken were centrifuged and resuspended in the same volume of the cell culture medium. MTS (3-(4,5-dimethylthiazol-2-yl)5-(3-carbox-ymethoxyphenyl)-2-(4-sulfophenyl)2 H-tetrazolium salt) reduction was measured as basal cytotoxicity endpoint following the protocol described by Cebadero et al. [27]. In order to show the correct performance of the cytotoxicity tests, additional experiments including a positive control (Triton X-100 0.3%) were carried out.

The tissues of the 3D model were treated with undigested rGO and digestas from the duodenal phase at different concentrations (25, 50, and 100 μg/mL) for 24 h. Triton X-100 (0.3%) was used as positive control. Cell viability was assayed by MTT (3-(4,5-dimethylthiazol-2-yl)-2,5-diphenyltetrazolium bromide) assay following the manufacturer’s protocol. After 24 h of exposure, the media was removed, and tissues were washed 3 times with phosphate buffer saline. Samples were moved to a 24-well plate with 300 µL of MTT solution. After 3 h of incubation, MTT was removed and 1 mL isopropanol was added. The plate was shaken in the dark for 1 h. An additional 1 mL of isopropanol was added, and the mixture was transferred to a 96-well plate. Absorbance was measured at 570 nm and % of viability was calculated.

### 2.6. Cytokines Detection

The inflammatory response of the different cell models after 24 h of exposure to undigested and digested rGO (undigested and duodenal phase in HepG2 and 3D model, and duodenal and colonic phases in Caco-2) were analyzed using a Bio-Plex PROTM human cytokine assay for the inflammatory markers interleukin 2 (IL-2), interleukin 6 (IL-6), interleukin 8 (IL-8), gamma interferon (INF γ), and tumor necrosis factor alpha (TNF-α). Lipopolysaccharide (LPS, from *Escherichia coli*, 1 µg/mL) was used as positive control. The supernatants were collected, centrifuged, and stored at −20 °C until analysis.

### 2.7. Statistical Analysis

Statistical analysis for viability in HepG2, Caco-2 cells, and the intestinal 3D model was carried out using one-way ANOVA, followed by Tukey’s multiple comparisons test for data with a normal distribution, and Kruskal–Wallis test followed by Dunn’s multiple comparison test for data that did not follow a normal distribution. All analyses were performed with GraphPad Prism 9 version 9.0.0 software. *p* values < 0.05 were considered significant. All experiments were performed at least three times (HepG2/Caco-2) and at least by triplicate per concentration.

## 3. Results and Discussion

### 3.1. Characterization of rGO after Digestion Process

**Figure 1:**
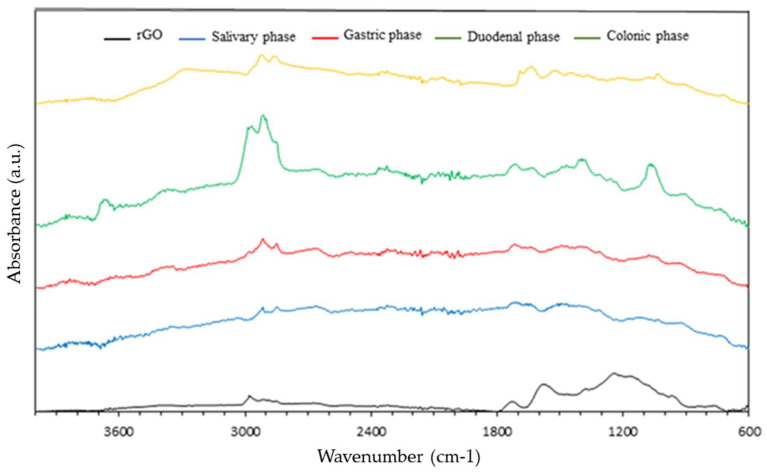
Comparison between FTIR spectra of undigested rGO and the different phases during the digestion process.

Figure 1 shows the FTIR spectrum of undigested rGO and rGO after different digestion phases. The FTIR spectrum of undigested rGO shows the characteristic peaks of the stretching vibration of the C-H groups around 2600–2800 cm^−1^. The slight peak at 1750 cm^−1^ is attributed to the stretching vibration of the C=O bonds. The absorption band at approximately 1600 cm^−1^ corresponds to the C=C bonding of aromatic rings within the rGO carbon skeleton structure. Other oxygenated functional groups observed in the undigested rGO spectrum include the -OH group at approximately 3400 and 1350 cm^−1^, C-OH around 1200 cm^−1^, and C-O at 1050 cm^−1^.

Regarding digested rGO samples, it should be noted that the peaks described above are maintained in all cases, although at a lower intensity. However, an increase in the intensity of the C-H absorption band is observed. This fact corroborates what was described by Bitounis et al. [18], who detailed that the agglomeration of carbonaceous material could favor associations between different regions of the g.i. system due to acidic conditions, bile salts, and other digestive enzymes, although with a lower intensity.

**Figure 2:**
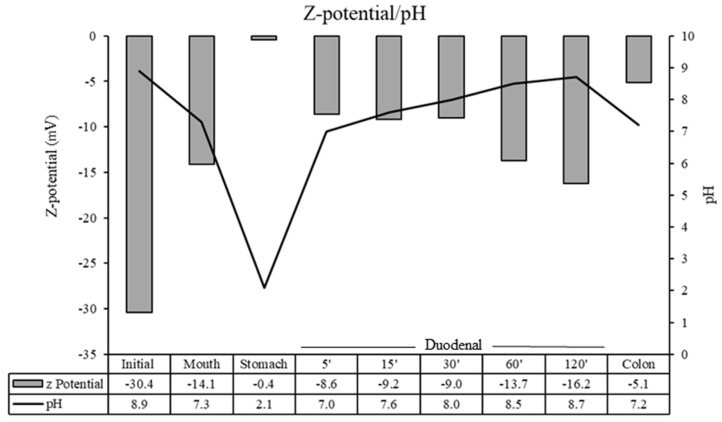
Z potential measurement of rGO and pH values in initial, saliva, gastric, duodenal, and colonic phases.

In order to determine the surface charge and colloidal stability of the tested solutions, the Z potential of the different samples was measured. Values around ±30 mV are considered as moderate stability. However, values close to zero may suggest a lower stability behavior [28]. The Z potential of rGO and pH values in the different phases are shown in Figure 2. The highest Z value observed was in undigested rGO (−30.4 mV) at pH 8.9, and the lowest value (−0.4) mV at the more acidic pH (2.1). These results confirm that the Z potential of rGO dispersions are pH-sensitive. Similar results were observed by Guarnieri et al. [17], which determined the stability of two GRM (FLG and GO) after simulated oral ingestion by Z potential spectroscopy. Both samples were unstable at low pH values (<5), forming agglomerates, and stable at pH values between 6.5 to 9. However, in another study, the authors observed that the acid treatment (pH < 2) did not affect the Z potential of GRM (GO and graphene nanoplatelet (GNP)) [16]. In addition, it is known that rGO forms stable dispersions in more basic media (pH 8–11.5), and its Z potential dispersions are pH-sensitive [29].

**Figure 3:**
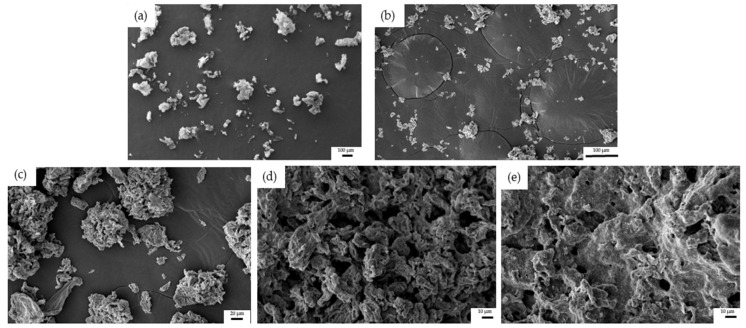
SEM images of 100 µg/mL rGO after (**a**) initial (bar = 100 µm), (**b**) saliva (bar = 100 µm), (**c**) gastric (bar = 20 µm), (**d**) duodenal (bar = 10 µm), and (**e**) colonic phases (bar = 10 µm).

In the initial and salivary phases (Figure 3a,b), rGO samples showed the lowest agglomeration. However, it increased in the following phases (gastric, duodenal, and colonic) (Figure 3c–e). Similarly, Bitounis et al. [18] used field emission scanning electron microscopy to study the morphological changes of small GO and micro GO. They observed agglomeration and morphological alterations of these materials during simulated digestion. Nevertheless, Kucki et al. [16] did not observe remarkable changes in the GO and GNP morphology by SEM images after acid treatment.

As mentioned above, all these changes in the morphology and agglomeration of GRM are supported by other authors and are attributed to the interaction with the conditions of the g.i. tract (acidic pH, bile salts, or digestive enzymes) [17,18,19]. Also, Guarnieri et al. [17] suggested that changes in the D band of Raman spectra observed in digested GO and FLG are related with their aggregation in large clusters in the g.i. tract.

### 3.2. Internalization and Cytotoxicity of Digested rGO in Different Cell Models

**Figure 4:**
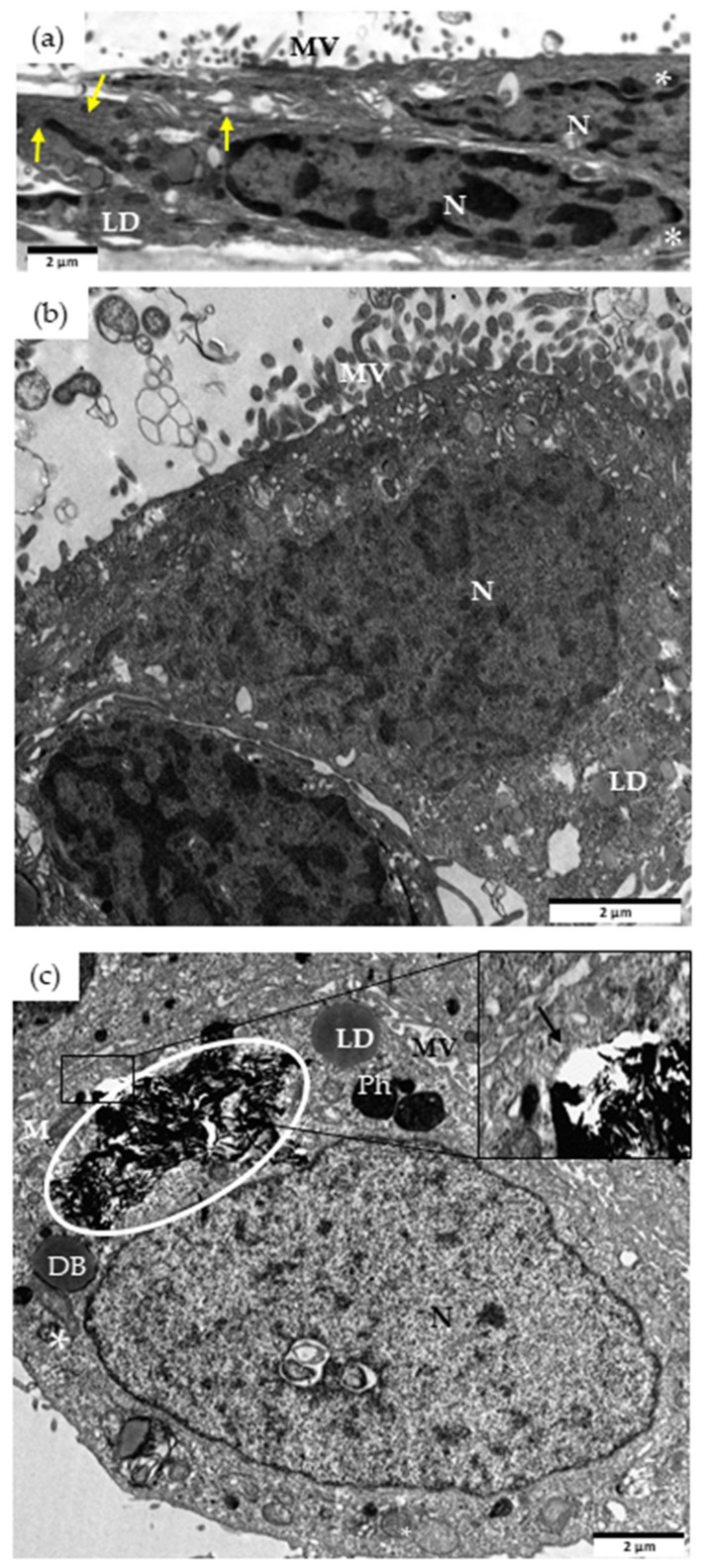
TEM images of cellular internalization of rGO in Caco-2 cells. Unexposed control cells (**a**), and cells exposed for 24 h to 100 µg/mL rGO from duodenal phase (**b**), or colonic phase (**c**). DB, dense bodies; Ph, phagosome; LD, lipid droplets; M, mitochondria; MV, microvilli; N, nucleus; *, multivesicular bodies; black arrow, endocytic membrane; yellow arrow, endoplasmic reticulum, and graphene materials indicated by the circle. Scale bar: 2 μm.

Internalization assays of undigested and digested rGO samples in Caco-2 are shown in Figure 4. Unexposed cells showed intact nuclei, cytosolic rough endoplasmic reticulum (RER) cisternae, free ribosomes, and isolated mitochondria (Figure 4). Caco-2 exposed to 100 µg/mL colonic rGO for 24 h showed internalization of graphene material (white circle) in endocytic vesicles (Figure 4c, inset). Phagosomes and dense bodies (probably RER dilations) are shown close to rGO (Figure 4c). No membrane rupture, altered mitochondrial cristae, nor apoptotic features were detected in Caco-2 exposed to colonic rGO. However, we did not detect internalized graphene material when cells were exposed to duodenal rGO for 24 h (Figure 4b). Moreover, Caco-2 cells did not show any cellular defects.

We have previously reported that undigested rGO was internalized by Caco-2 cells [27]. In relation to digested rGO, only Guarnieri et al. [17] have studied the cellular uptake of digested GRMs by Caco-2 cells after chronic incubation (9 days). They observed a limited internalization of GRMs, due to the large GRM aggregates that were accumulated on the cell membrane.

**Figure 5:**
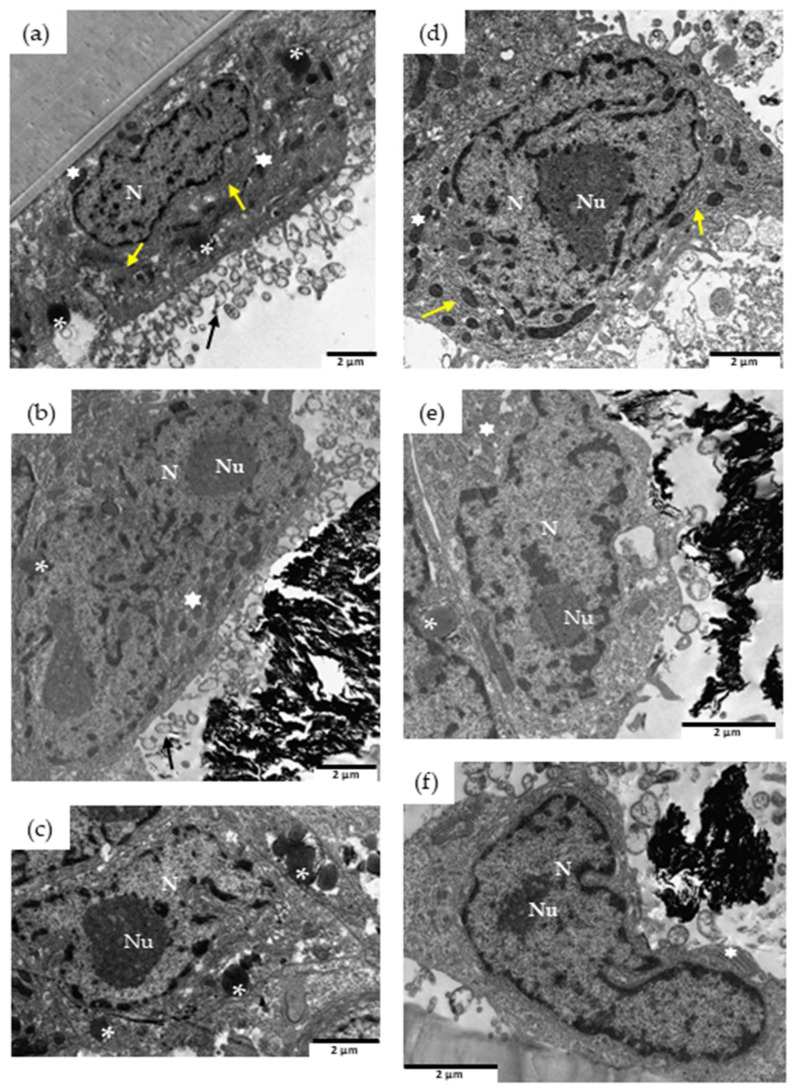
TEM images of cellular internalization of rGO in HepG cells for 24 h (**a**–**c**) and 48 h (**d**–**f**). Unexposed control cells (**a**,**d**), and cells exposed to 100 µg/mL undigested rGO (**b**,**e**), or digested rGO (**c**,**f**). N, nucleus; Nu, nucleolus; *, lipid droplets; yellow arrow, endoplasmic reticulum; black arrow, extracellular vesicles, and white star, mitochondria. Scale bar: 2 μm.

Unexposed HepG2 cells showed intact nuclei with a prominent nucleolus, cytosolic RER cisternae, several lipid droplets, isolated mitochondria with tubular cristae, a feature of cells with a high rate of lipid metabolism, and many extracellular vesicles (Figure 5a,d). HepG2 exposed to 100 µg/mL rGO, undigested or duodenal phase, did not internalize the graphene material (Figure 5b,c,e,f). Large graphene particles were found close to the outer leaflet of the cell membrane and no endocytic projections were found around graphene (Figure 5b,e,f). Moreover, no apoptotic nor necrotic cells were visualized and no alterations in nuclei nor any cellular organelles were found.

The number of studies that have assessed the internalization of rGO in HepG2 cells are very scarce. Chatterje et al. [30] reported that rGO (8 and 46 mg/L) was not uptaken by HepG2 cells after 24 h of exposure. These authors suggested that rGO was aggregated and accumulated in the cell membrane. In the present study, Z potential values and SEM images also showed an agglomeration of rGO due to the digestive process in comparison to the undigested sample. However, other GRM, such as GO, were internalized by this cell model [30,31]. These authors demonstrated that this material was uptaken by endocytosis processes and isolated into intracellular vesicles. The different results observed between GO/rGO could be attributed to the hydrophobic nature of rGO [30]. To our knowledge, this is the first study that has evaluated the internalization of GRM after a digestion process in HepG2 cells.

**Figure 6:**
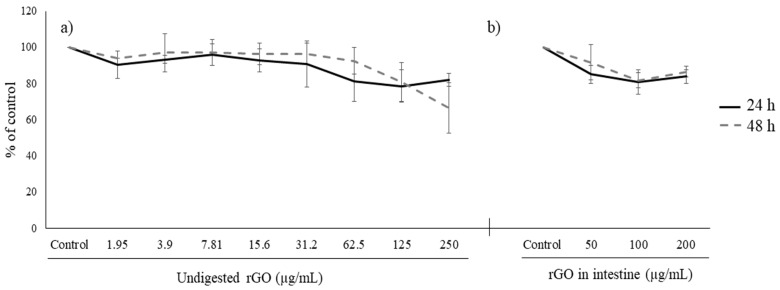
Viability of HepG2 cells exposed to 0–250 µg/mL undigested rGO (**a**), and 50, 100, and 200 µg/mL digested rGO (duodenal phase) (**b**) for 24 h and 48 h. All values are expressed as mean ± SD.

It is known that orally ingested toxicants can be metabolized in the g.i. tract [32], and they can be absorbed into the portal circulation causing hepatotoxicity [33]. Moreover, liver has been reported to accumulate nanoparticles and this may cause adverse effects in this organ [34]. For these reasons, we assayed the potential cytotoxic effects of undigested rGO and the digestas from the duodenal phase in HepG2 cells (Figure 6).

In Figure 6a, it can be observed that undigested rGO did not show a significant reduction in cell viability with respect to the control group after 24 and 48 h of exposure to any concentration assessed. Moreover, HepG2 cells exposed to rGO after the duodenal phase did not cause a significant reduction in viability under any of the conditions tested (Figure 6b). The adequate performance of the cytotoxicity tests was evidenced by the decrease observed in the positive control (Figure S1, Supplementary Material). The available results in the scientific literature reported a decrease in cell viability after exposure to rGO, however, these results are contradictory between them [30,35,36,37]. This is the case of Chartterjee et al. [30] and Ahamed et al. [36], who described a decrease in cell viability above 25 mg/L and 50 µg/mL, respectively. Moreover, Lingaraju et al. [35] observed a concentration-dependent cytotoxicity leading to a median inhibitory concentration (IC50-24 h) of 357.53 µg/mL, and Zuchowska et al. [37] observed cytotoxicity at all tested concentrations (200–800 µg/mL), although they were higher than in the present work. The different cytotoxicity effects observed could be due to its physicochemical properties, such as size, shape, aggregation state, and its interactions with cells [38]. However, there are no previous studies on the effects of digested rGO or any other digested GRM in this cell model [39].

**Figure 7:**
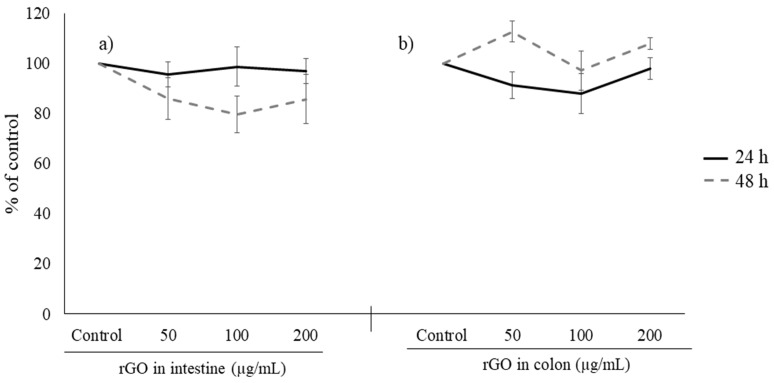
Reduction of tetrazolium salt (**a**,**b**) of Caco-2 cells after 24 h and 48 h of exposure to 50, 100, and 200 μg/mL digested rGO (duodenal phase) (**a**) and digested rGO (colonic phase) (**b**). All values are expressed as mean ± SD.

**Figure 8:**
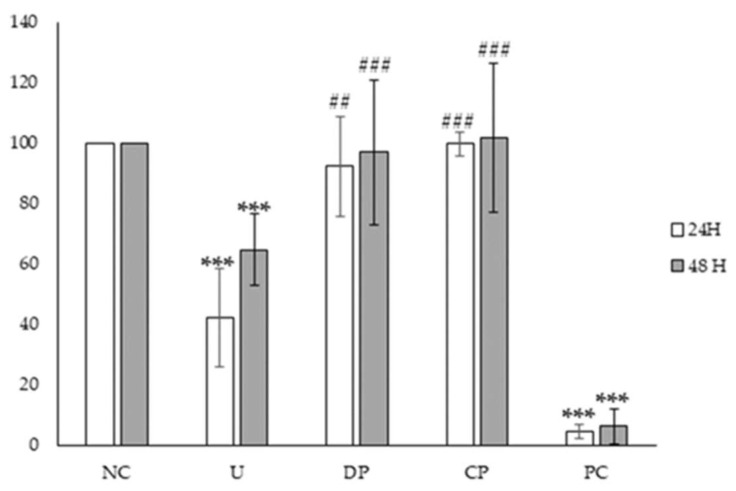
Viability of Caco-2 cells after 24 and 48 h exposure to negative control (NC), duodenal phase 200 µg/mL (DP), colonic phase 200 µg/mL (CP), undigested rGO 200 µg/mL (U), and positive control (Triton X-100 0.3%, PC). Values expressed as mean ± sd. ***, *p* < 0.001 significantly different from the negative control group; ##, *p* < 0.01, ###, *p* < 0.001 significantly different from the undigested sample.

As we mentioned above, Caco-2 is extensively used as an intestinal epithelium model because it expresses morphological and functional characteristics of enterocytes [25]. For this reason, we have used this cell line to investigate the cytotoxicity of duodenal and colonic phases. Previously, we reported that undigested rGO reduced cell viability in a significant way using MTS reduction assay leading to a mean effective concentration (EC50) value of 176.3 ± 7.6 µg/mL for 24 h [27]. However, in this study, no evident cell viability decrease was observed with digested rGO after duodenal and colonic phases (Figure 7a,b) whereas positive control showed a significant decrease (Figure S1). Moreover, when the same concentration of undigested and digested samples was tested, cytotoxicity was only observed with undigested rGO, with significant differences compared to digested ones (Figure 8). Our results suggest that the digestion process has prevented the cytotoxicity observed. However, other authors such as Kucki et al. [16] observed that control and acid-treated GO and GNP did not affect cell viability in undifferentiated Caco-2 cells, so the acid treatment did not have an impact on cytotoxicity. Guarnieri et al. [17] found that digested GRM (FLG and GO) were well tolerated by the intestinal barrier and did not induce its disruption/perturbation upon chronic exposure (up to 9 days), but they did not test the non-digested counterparts. Bitounis et al. [18] used a triculture cell model (Caco-2, HT29-MTX, and Raji-B cells) to assess the cytotoxicity of small intestinal digestas of submicron- and micron-sized GO (1 and 5 µg/mL). They did not observe a decrease in their cell viability, with no data on undigested samples, but they detected an increase in reactive oxygen species (ROS) levels. On the other hand, Bazina et al. [19] studied the cytotoxicity of digested GRM (small, medium, and large GO, and small and large rGO and partially rGO) by different assays using the same triculture cell model. These authors observed a slight decrease in cell viability (by mitochondrial enzymatic activity) after exposure to small rGO (5 µg/mL), as well as a light increase in LDH release after exposure to large and small rGO (1 µg/mL). The toxicity of graphene in eukaryotic cells depends on several factors, such as chemical composition, layer number, size, shape, and charge [40]. Hence, the lack of cytotoxicity observed in Caco-2 cells after exposure to digested rGO in comparison to undigested rGO [27] could be due to the agglomeration of the particles in the duodenal and colonic phases.

**Figure 9:**
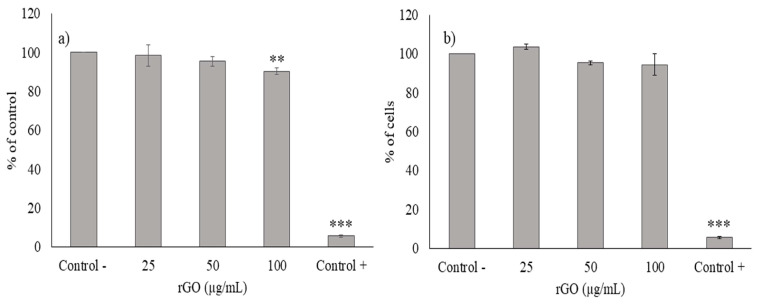
Cytotoxicity of undigested rGO (**a**), and digested rGO (duodenal phase) (**b**) at different concentrations (0–100 µg/mL) after 24 h in the EpiIntestinal^TM^ model. All values are expressed as mean ± SD. Triton (0.3%) was used as positive control. **, *p* < 0.01 and ***, *p* < 0.001 significantly different from the control group.

Besides enterocytes, other types of cells, such as paneth cells, M cells, tuft cells, and intestinal stem cells constitute the intestinal epithelium. In this sense, 3D models provide more realistic tissue response in comparison with the Caco-2 2D culture [41]. The main advantage of these models in toxicology in comparison to 2D models, is to obtain results more similar to human tissues, and therefore to avoid the use of animals [42]. In this work, the results obtained showed a slight but statistically significant decrease (*p* < 0.01 **) on viability at 100 µg/mL undigested rGO (Figure 9). The cytotoxicity observed in the 3D intestinal model was less pronounced in comparison to that observed in Caco-2 cells after rGO exposure [27], which suggests that this 3D model is more resistant to the toxic insult. This agrees with the findings of Kucki et al. [43] who observed that differentiated Caco-2 cultures did not uptake GO but not-differentiated cultures did. On the other hand, digested rGO did not cause any effect on viability at any concentration tested in this model. This suggests that digestion prevents the cytotoxic effects induced by rGO. The agglomeration of rGO through the different g.i. phases could be related to the lack of cytotoxicity observed. Bruinink et al. [44] described that an increase of the agglomeration of engineered nanoparticles (as induced by digestion) decreased cytotoxicity.

### 3.3. Cytokines Detection

Cytokine levels (pg/mL) were measured in the supernatants of the different cell cultures used in this study. With respect to HepG2 and Caco-2 cells, all values showed no detectable ranges of all cytokines measured (Table S2). In relation to the 3D model, the IL-1α neither exhibit detectable ranges. In the case of HepG2, some authors also reported that this cell line did not secrete TNF-α, IL-1β, or IL-6 in a basal way. However, substances such as ethanol, acetaldehyde, or LPS could increase the secretion of these cytokines [45]. In concordance with our results, Guarnieri et al. [17] also observed an absence of release of inflammatory cytokines such as IL-1β, IL-6, INF-γ, or TNF-α after chronic exposure to digested GRMs in Caco-2 cells. However, other inflammatory cytokines, such as IL-8 and MCP-1, were found. In this case, the digested GRMs did not induce any response.

### 3.4. General Discussion

From the results obtained, diverse observations can be derived. Thus, digested rGO up to 200 µg/mL induced a low toxicity in the different cellular models employed. These models showed a different sensitivity to GRM, and HepG2 is less sensitive to rGO in comparison to Caco-2 cultures, and those are more sensitive than 3D intestinal cultures. Also, the digestion process seems to have a protective effect, which can be in part due to the changes in physicochemical properties of rGO that promote agglomeration (as evidenced by Z potential values and SEM images in comparison to the undigested sample), and therefore a reduced uptake by the cells. However, in this case (as well as in the scientific literature), a fasted in vitro digestion model has been used, and therefore potential interactions with the food matrix have not been taken into account. In any case, digestion cannot totally prevent uptake and systemic effects of GRM after oral exposure, as different reports have already evidenced in mammalian models [46,47,48], but this is a point to take into account in view of the risk assessment of GRM. Moreover, other aspects regarding local toxicity, and the analysis of more sensitive parameters such as molecular effects (transcriptomic, proteomic) or impact on microbiota [49,50] should be considered to elucidate the real intestinal toxicity of GRM.

## 4. Conclusions

Digested rGO samples did not induce evident toxicity (neither cytotoxicity nor inflammatory response) in HepG2, Caco-2, and 3D intestinal models at the conditions assayed. This lack of effects can be due to the increased agglomeration of rGO associated with the digestion process that could hamper the uptake by the cells or modify the rGO cellular interaction. Additional studies on the topic would be of interest to contribute to the human risk assessment of GRM after oral exposure.

## Data Availability

Data will be made available upon request.

## Supplementary Materials

The following supporting information can be downloaded at: https://www.mdpi.com/article/10.3390/nano13162285/s1, Figure S1: Viability of (a) Caco-2 and (b) HepG2 cells after 24 h exposure to negative control (NC), duodenal phase 200 µg/mL (DP), colonic phase 200 µg/mL (CP), undigested rGO 250 µg/mL (U) and positive control (Triton X-100 0.3%, PC). Values expressed as mean ± sd. *** P < 0.001 significantly different from the negative control group; Table S1: Studies available in the scientific literature in relation to cytotoxicity of graphene materials subjected to an in vitro digestion procedure; Table S2: Cytokine levels (pg/mL) in the supernatants of Caco-2 and HepG2 cells after exposure to 100 µg/mL undigested and digested rGO samples for 24 h.
