# Supplementary material for: Impact of Gastrointestinal Digestion In Vitro Procedure on the Characterization and Cytotoxicity of Reduced Graphene Oxide
Source: Nanomaterials (Basel). 2023 Aug 9;13(16):2285. doi: 10.3390/nano13162285 (PMC10457766; doi:10.3390/nano13162285)
Supplement: Supplementary file 1 [file nanomaterials-13-02285-s001.zip › nanomaterials-2482267-supplementary.pdf]

nanomaterials

MDPI

Supplementary Materials

Impact of Gastrointestinal Digestion In Vitro Procedure on the Characterization and Cytotoxicity of Reduced Graphene Oxide

Óscar Cebadero-Domínguez [1], Leticia Diez-Quijada [1], Sergio López [2], Soraya Sánchez-Ballester [3], María Puerto [1], Ana M. Cameán [1] and Angeles Jos [1,*]

1 Area of Toxicology, Faculty of Pharmacy, Universidad de Sevilla, 41012 Seville, Spain; ocebadero@us.es (Ó.C.-D.); ldiezquijada@us.es (L.D.-Q.); mariapuerto@us.es (M.P.); camean@us.es (A.M.C.)
2 Department of Cell Biology, Faculty of Biology, Universidad de Sevilla, 41012 Seville, Spain; serglom@us.es
3 Packaging, Transport and Logistic Research Institute, Albert Einstein, 1, Paterna, 46980 Valencia, Spain; soraya.sanchez@itene.com
* Correspondence: angelesjos@us.es

Table S1. Studies available in the scientific literature in relation to cytotoxicity of graphene materials subjected to an in vitro digestion procedure.

Material	Physicochemical properties	Experimental Model	Relevant Results	Reference
GO, GNP	Size (µm), thickness, C/O ratio, ζ potential (mV): GO (1-40, 0.7-1.2 nm, 1.7, -39.4 ± 1.3 mV), GNP (1-10, 5 µm, 24, -62.6 ± 1.9)	*In vitro* digestion in acidic conditions (Incubation with 0.1 M HCl for 2 h)	The acid-treatment in both graphene derivatives did not produce changes in the material properties. No differences were observed in the viability of Caco-2 cells exposed to water or acid-treated graphenes.	Kucki et al., 2016
FLG, GO	Flake length (nm), ζ potential (mV): FLG (400, -15.8 ± 6.65) GO (400, -35.7 ± 7.69)	*In vitro* digestion assay (mouth, stomach and small intestine)	A modulation of the aggregation state of FLG and GO after *in vitro* digestion was observed. No structural changes or degradation were reported. The digested materials did not cause cytotoxicity or inflammatory response in Caco-2 intestinal layer after chronic exposure.	Guarnieri et al., 2018
GO (sub-micron and micron sizes	Lateral size (µm), C/O ratio, ID/IG ratio: smGO (0.24, 1.37, 0.83), mGO (1.13, 1.41, 0.84)	Fasting food models (oral, gastric and small intestinal phases)	smGO and mGO samples presented an increase in agglomeration upon simulated gastrointestinal digestion. A substitution of the oxygen on the surface with nitrogen-containing group was observed. No cytotoxic effects were observed in an tri-culture small intestinal model after exposure to GO small intestinal digestas. Both materials increased reactive oxygen species (ROS) production at 5 µg/mL compared to control.	Bitounis et al., 2020
GO-small, medium and large, prGO, rGO-small and large	Lateral size (nm), thickness (nm) , C/O ratio: GO-S (271 ± 34, 0.77 ± 0.08; 64:35), GO-M (462 ± 114, 0.94 ± 0.25, 61:39), GO-L (1560 ± 750, 0.97 ± 0.25, 61:39), prGO (357 ± 42, 3, 72:28), rGO-S (411 ± 79, 2.25 ± 1.85, 78:22) rGO-L (2015 ± 674, 62, 78:22)	*In vitro* simulated digestion (oral, gastric and small intestinal digestion)	An overall shift to larger sizes was observed in the case of GO after simulated digestion. rGO materials caused slight increase in lactate deshydrogensase (LDH) leakage compared to controls. Digestas of rGO-s produced a decrease on cell viability (mitochondrial enzymatic activity). No changes in caspase 3/7 activity were observed after exposure to digested materials. Cells exposed to GO-s and GO-l showed an increase of ROS generation.	Bazina et al., 2021

FLG: few-layer graphene; GO: Graphene oxide; GNP: Graphene nanoplatelets; HCl: hydrochloric acid; prGO: partially reduced graphene oxide; rGO (reduced graphene oxide), S (small).

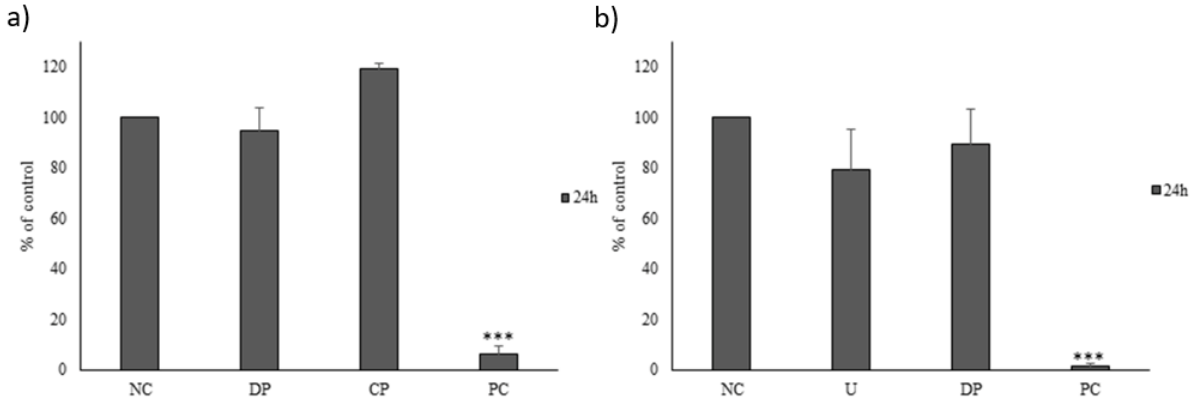

Figure S1. Viability of (a) Caco-2 and (b) HepG2 cells after 24h exposure to negative control (NC), duodenal phase 200 µg/mL (DP), colonic phase 200 µg/mL (CP), undigested rGO 250 µg/mL (U) and positive control (Triton X-100 0.3%, PC). Values expressed as mean ± sd. *** $p < 0.001$ significantly different from the negative control group.

Table S2. Cytokine levels (pg/mL) in the supernatants of Caco-2 and HepG2 cells after exposure to 100 µg/mL undigested and digested rGO samples for 24h.

Cell line	Caco-2					HepG2				
	Mean ± SD (pg/mL)					Mean ± SD (pg/mL)				
	IL-1B	IL-2	IL-6	TNF-α	IFN-ϒ	IL-1B	IL-2	IL-6	TNF-α	IFN-ϒ
Control -	ND	ND	3.13±0.5	ND	ND	ND	ND	ND	ND	ND
Undigested	ND	0.74±0.6	3.28±0.4	ND	ND	ND	ND	ND	ND	ND
Duodenal phase	ND	ND	3.78±0.6	ND	ND	ND	ND	ND	ND	ND
Colonic phase	ND	ND	3.76±0.6	ND	ND	--	--	--	--	--
Positive Control (LPS)	ND	ND	5.47±0.7*	ND	ND	ND	ND	ND	ND	ND

ND: not detected; --: not measured.
